# Early childhood exposure to secondhand smoke and behavioural problems in preschoolers

**DOI:** 10.1038/s41598-018-33829-6

**Published:** 2018-10-18

**Authors:** Tzu Tsun Luk, Man Ping Wang, Yi Nam Suen, David Soo-quee Koh, Tai Hing Lam, Sophia Siu-chee Chan

**Affiliations:** 1 School of Nursing, The University of Hong Kong, Pok Fu Lam, Hong Kong; 2 Department of Psychiatry, The University of Hong Kong, Pok Fu Lam, Hong Kong; 3 Saw Swee Hock School of Public Health, National University of Singapore, Singapore, Singapore; 4 PAPRSB Institute of Health Sciences, Universiti Brunei Darussalam, Gadong, Brunei; 5 School of Public Health, The University of Hong Kong, Pok Fu Lam, Hong Kong

## Abstract

Evidence on behavioural abnormalities in children exposed to secondhand smoke is limited. This study examined the relation between infant/ toddler cotinine concentration, a biomarker of secondhand smoke exposure, and behavioural problems in preschoolers who were unexposed to maternal smoking during gestation. A prospective cohort of 301 non-smoking mothers with their young children aged ≤18 months visiting postnatal primary care clinics in Hong Kong was enrolled in 2012 and followed by telephone survey 3 years afterwards. Saliva was collected at baseline for cotinine assay. Child behavioural health at 3-year follow-up was assessed by the parent-reported Strengths and Difficulties Questionnaire (SDQ). We conducted multivariable linear regressions to compute regression coefficients (*b*) of SDQ scores in relation to salivary cotinine level. Mean ± SD age of children at follow-up was 3.7 ± 0.5 years and 50.8% were boys. After adjusting for age, sex, birthweight, household income, housing type, maternal education and depressive symptoms, greater cotinine concentrations during early childhood were associated with greater conduct problems (*b* = 0.90, 95% CI 0.03–1.76) and hyperactivity/ inattention (*b* = 1.12, 95% CI 0.07–2.17) at preschool age. This study corroborates previous findings on the potential role of secondhand smoke in development of child behavioural problems.

## Introduction

Despite growing literature on the associations of secondhand smoke (SHS) exposure with behavioural problems in children, evidence is inadequate to infer causality^1,2^. Recent reviews suggested SHS exposure may contribute to development of hyperactivity/ inattention and conduct problems in children but did not identify any prospective study that used an objective measure of SHS (e.g. cotinine)^3,4^. Many studies were also limited by suboptimal control for maternal smoking during pregnancy (MSP)^5^, which may have independent effect on child behavioural problems^6^. Given up to 40% of the world’s children are exposed to SHS^7^, more robust evidence on its harm is imperative to advocate for stronger tobacco control measures.

It is challenging to separate the effect of postnatal SHS exposure from MSP on child health outcomes since mothers who smoked during pregnancy are likely to continue smoking postnatally^2^. Statistical control for MSP (e.g. by multivariable regression) may yield misleading estimates due to high collinearity between prenatal and postnatal maternal smoking^5^. A better approach is to restrict the analysis to offspring of non-smoking mothers^5^. However, among the few studies that followed the recommended approach, one was cross-sectional^8^ and the others relied on subjective maternal report of child SHS exposure^9,10^, which is prone to underestimation and potentially biased^11,12^.

Hong Kong is a densely-populated city where MSP is rare (3.4%)^13^, but SHS is pervasive due to the crowded living environment and multiunit housing^14^. An increased exposure to SHS in children at home was observed after smoking was outlawed in most public areas in 2007^15^. We were not aware of any study on SHS exposure in children aged ≤18 months, who typically reside at home most of the time, and subsequent risk of behavioural problems. Therefore, using a prospective cohort of young children whose mothers were non-smokers in Hong Kong, we examined the association of SHS exposure during early childhood with behavioural health at preschool age.

## Methods

### Study design

A cross-sectional study was conducted from April to August 2012 in 4 of the 33 Maternal and Child Health Centres (MCHC) in Hong Kong, which provide child health assessments and immunisation services to the general public. Details of the baseline study have been reported elsewhere^16^. Nurses on duty in the MCHC screened the eligibility of mothers with their children based on the following criteria: (1) the mother is a non-smoker; (2) the child is under 18 months; (3) the parents and the child live together in the same household; (4) the mother is able to communicate in Cantonese/Putonghua; and (5) both the mother and the child are Hong Kong residents. A total of 692 non-smoking mothers with an infant/toddler (mean ± SD age 6.2 ± 5.7 months) completed a self-administered questionnaire on their child’s health and family socio-demographics and provided contact information. In 2015, an *a posteriori* telephone follow-up survey was conceived 3 years after the baseline study, which included measures of child behavioural problems using the Strengths and Difficulties Questionnaire (SDQ).

The study protocol was approved by the Institutional Review Board of the University of Hong Kong/Hospital Authority West Cluster. The conduct of the study follows the Declaration of Helsinki. Informed consent was obtained from all mothers prior to participation.

### Measures

Cotinine, a major metabolite of nicotine, is the biomarker of choice to quantify SHS exposure^17^. We collected saliva as it can be sampled easily and non-invasively relative to urine and blood sampling from young children. To collect saliva, trained research assistants placed a sorbette (a sponge-tip shaft) under the child’s tongue for 15 to 30 seconds. The sorbette soaked with saliva was immediately stored in a 2 mL microcentrifuge tube and then frozen at −20 °C using ice pads in a cooler box for transportation. Salivary cotinine concentrations (ng/mL) were analysed at the National University of Singapore using an enzyme-linked immunosorbent assay kit (Salimetrics), which has a minimum detectable limit of 0.05 ng/mL. Salivary samples were also obtained from the mothers to verify their non-smoking status.

The child’s behavioral health at the 3-year follow-up was assessed using 25 items on psychological attributes of the parental-report version of the SDQ^18^, which has been validated locally^19^. The 25 items are divided into 5 subscales: emotional symptoms, conduct problems, hyperactivity/inattention, peer relationship problems and prosocial behaviour. Each item has a 3-point rating scale of 0 to 2, which are summed to give an overall score ranging from 0 to 10 for each subscale. The total difficulties score is the sum of all but the prosocial behaviour subscales. The parental-report version of the SDQ has also been validated as a screening tool for attention deficit/hyperactivity disorder (ADHD) and conduct disorder in preschoolers at ages 3 to 4 years^20^.

Potential confounders that may influence both SHS exposure and child behavioural problems were also assessed. At baseline, we recorded the date of birth, sex and birth weight (kg) of the children, maternal education level, monthly household income and housing type. At follow-up, we assessed maternal depressive symptoms using the Patient Health Questionnaire-9 (PHQ-9), which has been validated in the local Chinese general population^21^. Higher composite scores in the PHQ-9 (range 0–27) indicate greater severity of depressive symptoms.

### Statistical analyses

Cotinine concentration was analysed as a continuous variable with logarithmic-transformation (*log*_10_) because of its skewed distribution, which is also a standard method used in previous studies^8,22^. Baseline characteristics of subjects with complete data on cotinine and SDQ were compared with those without complete data on cotinine and SDQ using t-test, Mann-Whitney U test and chi-square test as appropriate. Since SDQ scores were normally distributed, linear regressions were used to examine the associations of sample characteristics with SDQ subscale scores and total difficulties scores.

The associations between SDQ scores and log-transformed salivary cotinine concentration were also examined using linear regressions. A regression coefficient (*b*) of 1.00 means the SDQ scores increase by 1 unit with 10 times increase in salivary cotinine concentration. In addition to child’s age and sex, potential confounders including birthweight, maternal education, household income and housing type were adjusted in model 1^23,24^. Few previous studies on SHS and child behavioural health controlled for maternal mental health^25,26^. In model 2, we further adjusted for maternal depressive symptoms (PHQ-9)^23,24^. As a sensitivity test, we repeated the multivariable regression analyses with exclusion of infants whose mothers had salivary cotinine concentration of ≥12 ml ng/mL, which suggests active smoking^27^.

Incomplete data on log-transformed infant salivary cotinine (n = 112) were imputed using multivariate normal imputation on a missing at random assumption^28^. The imputation model included all potential confounders and outcome variables^29^. Fifty datasets were created. The distributions of the imputed data compared well to the observed data as assessed by diagnostic plots^30^, which were corroborated by the results of Kolmogorov-Smirnov tests (*P* < 0.05 only in 2 out of the 50 imputed data sets). The mean ± SD log-transformed cotinine level of the imputed data was also very similar to that of the observed data (0.0489 ± 0.45 vs 0.0485 ± 0.38). Complete case analysis was also performed. All analyses were conducted using Stata/IC version 13.1 with 2-sided *P* < 0.05 denoting statistical significance.

## Results

Of the 692 mother-child pairs recruited at baseline, 445 (64.3%) mothers were successfully re-contacted to participate in the telephone follow-up survey. Loss to follow-up were mostly due to failure to contact (n = 177) and refusal (n = 66). The present study analysed data collected from 301 mother-infant dyads who completed the SDQ, which was included in the follow-up questionnaire as an optional component. Of these 301 dyads, 189 provided valid infant saliva sample for cotinine assay at baseline. The unavailability of cotinine data was mostly due to insufficient saliva sample collected by the sorbette for cotinine assay, especially in younger children (5.7 vs 6.2 months old).

Table 1 shows that subjects with (n = 189) or without (n = 503) complete data on cotinine and SDQ were similar with regard to all baseline characteristics including salivary cotinine concentration, age, sex, birthweight, household monthly income, housing type and maternal education (all *P* ≥ 0.11; effect sizes ≤0.094). The analytic sample (subjects with data in SDQ; n = 301) were also similar to the excluded sample (n = 391) in terms of all baseline characteristics (*P* = 0.11 to 0.68; effect sizes 0.016 to 0.163).

**Table 1:** Baseline characteristics of the Chinese infants by completeness of data on cotinine and SDQ.

	Subjects with complete data for cotinine and SDQ (n = 189)		Subjects without complete data for cotinine and SDQ (n = 503)		*P* value^a^	Effect size
	N	(%)	N	(%)		
Mean (SD) *log*_10_-cotinine level, ng/ml	0.049	(0.38)	0.062	(0.39)	0.72	0.036
Mean (SD) age, month	6.2	(5.0)	6.7	(6.0)	0.68	0.085
Mean (SD) birthweight, kg	3.17	(0.46)	3.14	(0.59)	0.59	0.046
Sex					0.89	0.005
Boys	97	(51.3)	261	(51.9)		
Girls	92	(48.7)	242	(48.1)		
Monthly household income (HK$)^b^					0.24	0.078
≤$9999	14	(7.4)	57	(11.4)		
$10000–19999	54	(28.6)	159	(31.8)		
$20000–29999	45	(23.8)	115	(23.0)		
≥$30000	76	(40.2)	169	(33.8)		
Housing type					0.12	0.093
Private housing	80	(42.3)	200	(39.9)		
Subsidized sale flats	42	(22.2)	80	(16.0)		
Public rental housing	62	(32.8)	200	(39.9)		
Others	5	(2.7)	21	(4.2)		
**Maternal education**					0.50	0.045
Junior secondary or below	33	(17.5)	100	(20.0)		
Senior secondary	90	(47.6)	248	(49.5)		
Tertiary	66	(34.9)	153	(30.5)		

^a^Computed by t test, Mann-Whitney U test and Pearson χ2 test as appropriate. ^b^HK$7.8 ≈ US$1.

The children stayed at home most of time at baseline (mean ± SD hours per day in the past week = 21.5 ± 3.8). At the 3-year follow-up, the mean ± SD age of children was 3.7 ± 0.5 years and 50.8% were boys. Most mothers (n = 292/301) reported none to minimal depressive symptoms (PHQ-9 score ≤4) with mean ± SD score of 0.8 ± 1.6. Table 2 shows that maternal depressive symptoms were strongly associated with hyperactivity/ inattention, peer relationship problems and total difficulties of the children.

**Table 2:** Bivariable associations between sample characteristics and SDQ scores in Chinese preschoolers (n = 301).

	N	(%)	Coefficient (95% confidence interval)^a^					
			Emotional symptoms	Conduct problems	Hyperactivity/ inattention	Peer relationship problems	Prosocial behaviour	Total difficulties
Mean (SD) age at follow-up, year	3.70	(0.50)	−0.04 (−0.38, 0.29)	−0.11 (−0.59, 0.37)	−0.18 (−0.71, 0.35)	−0.16 (−0.46, 0.13)	0.44 (−0.20, 1.07)	−0.44 (−1.66, 0.80)
Mean (SD) birthweight, kg	3.17	(0.47)	0.25 (−0.10, 0.61)	0.33 (−0.18, 0.84)	−0.01 (−0.57, 0.56)	**−0.35 (−0.66, −0.03)**	0.07 (−0.61, 0.75)	0.26 (−1.05, 1.57)
Sex								
Boys	153	(50.8)	Ref.	Ref.	Ref.	Ref.	Ref.	Ref.
Girls	148	(49.2)	0.02 (−0.32, 0.35)	−0.24 (−0.72, 0.24)	−0.04 (−0.57, 0.49)	−0.12 (−0.42, 0.17)	0.36 (−0.28, 0.99)	−0.43 (−1.66, 0.79)
Monthly household income (HK$)^b^								
≤$9999	24	(8.0)	Ref.	Ref.	Ref.	Ref.	Ref.	Ref.
$10000–19999	91	(30.2)	0.20 (−0.46, 0.86)	0.14 (−0.81, 1.10)	**1.33 (0.29, 2.36)**	−0.15 (−0.74, 0.43)	0.53 (−0.74, 1.79)	1.45 (−0.98, 3.87)
$20000–29999	73	(24.3)	0.25 (−0.43, 0.92)	0.43 (−0.55, 1.41)	1.00 (−0.06, 2.06)	−0.29 (−0.89, 0.31)	0.76 (−0.53, 2.06)	1.39 (−1.10, 3.87)
≥$30000	113	(37.5)	0.11 (−0.53, 0.76)	0.18 (−0.76, 1.11)	0.77 (−0.24, 1.79)	−0.43(−1.00, 0.15)	0.61 (−0.63, 1.84)	0.57(−1.81, 2.94)
Housing type								
Private housing	116	(38.5)	Ref.	Ref.	Ref.	Ref.	Ref.	Ref.
Subsidized sale flats	65	(21.6)	−0.16 (−0.60, 0.28)	−0.22 (−0.87, 0.43)	−0.31 (−1.01, 0.40)	0.09 (−0.31, 0.48)	−0.28 (−1.14, 0.58)	−0.74 (−2.39, 0.90)
Public rental housing	110	(36.5)	0.14 (−0.25, 0.52)	0.14 (−0.42, 0.69)	−0.05 (−0.66, 0.56)	**0.37 (0.03, 0.71)**	0.17 (−0.57, 0.90)	0.56 (−0.85, 1.97)
Others	10	(3.3)	−0.73 (−1.67, 0.21)	−0.22 (−1.59, 1.15)	−0.82 (−2.32, 0.68)	−0.70 (−1.53, 0.13)	0.65 (−1.16, 2.47)	−2.47 (−5.93, 0.99)
Maternal education								
Junior secondary or below	54	(17.9)	Ref.	Ref.	Ref.	Ref.	Ref.	Ref.
Senior secondary	146	(48.5)	−0.32 (−0.78, 0.13)	−0.01 (−0.66, 0.65)	0.04 (−0.68, 0.76)	−0.12 (−0.53, 0.28)	0.35 (−0.53, 1.24)	−0.49 (−2.16, 1.17)
Tertiary	101	(33.6)	**−0.61 (−1.09, −0.13)**	−0.67 (−1.37, 0.02)	−0.68 (−1.44, 0.08)	**−0.46 (−0.89, −0.03**)	0.57 (−0.36, 1.51)	**−2.43 (−4.19, −0.68)**
Maternal depressive symptom^c^								
Minimal	413	(94.3)	Ref.	Ref.	Ref.	Ref.	Ref.	Ref.
Mild	22	(5.0)	0.62 (−0.48, 1.71)	0.67 (−0.91, 2.26)	**1.98 (0.26, 3.69)**	0.45 (−0.51, 1.42)	−1.17 (−3.27, 0.94)	3.77 (−0.21, 7.74)
Moderate	3	(0.7)	1.40 (−0.63, 3.44)	1.81 (−1.13, 4.75)	**3.76 (0.58, 6.94)**	**2.60 (0.81, 4.38)**	0.91 (−2.99, 4.80)	**9.62 (2.25, 17.0)**

^a^Bold face indicates *P < *0.05. ^b^HK$7.8 ≈ US$1. ^c^Assessed by Patient Health Questionnaire-9.

Table 3 shows that after adjusting for age, sex, birthweight, monthly household income and maternal education (model 1), higher cotinine concentration were associated with higher scores in conduct problems (*b* = 0.93, 95% CI 0.07 to 1.78) and hyperactivity/ inattention (*b* = 1.27, 95% CI 0.22 to 2.32) subscales. The associations were attenuated but remained statistically significant after further adjusting for maternal depressive symptoms (model 2). No significant association was observed between salivary cotinine level and other SDQ domains in the adjusted models.

**Table 3:** Association of log_10_-transformed infant salivary cotinine (ng/ ml) with Strengths and Difficulties Questionnaire (SDQ) scores in Chinese preschoolers.

	Mean (SD)SDQ score	Coefficient (95% confidence interval)^a^		
		Crude	Model 1^b^	Model 2^c^
**Imputed data analyses (n** = **301)**				
Emotional symptoms	1.1 (1.5)	0.11 (−0.44, 0.66)	0.05 (−0.57, 0.66)	−0.00 (−0.62, 0.62)
Conduct problems	2.2 (1.5)	**0.91 (0.14, 1.67)**	**0.93 (0.07, 1.78)**	**0.90 (0.03, 1.76)**
Hyperactivity/inattention	3.8 (2.3)	**1.19 (0.25, 2.13)**	**1.27 (0.22, 2.32)**	**1.12 (0.07, 2.17)**
Peer relationship problems	2.4 (1.3)	0.12 (−0.38, 0.61)	0.04 (−0.52, 0.60)	−0.03 (−0.58, 0.51)
Prosocial behaviour	5.6 (2.8)	−0.24 (−1.30, 0.81)	−0.14 (−1.30, 1.02)	−0.10 (−1.28, 1.09)
Total difficulties	9.5 (5.4)	**2.33 (0.19, 4.46)**	2.31 (−0.07, 4.70)	1.99 (−0.37, 4.35)
**Complete case analyses (n** = **189)**				
Emotional symptoms	1.1 (1.4)	0.21 (−0.33, 0.75)	0.13 (−0.44, 0.70)	0.05 (−0.52, 0.62)
Conduct problems	2.1 (2.0)	0.68 (−0.06, 1.41)	0.68 (−0.11, 1.47)	0.76 (−0.02, 1.54)
Hyperactivity/inattention	3.8 (2.3)	**1.08 (0.14, 2.01)**	**1.18 (0.20, 2.16)**	**1.02 (0.06, 1.99)**
Peer relationship problems	2.5 (1.3)	0.14 (−0.40, 0.68)	−0.00 (−0.55, 0.56)	−0.00 (−0.57, 0.57)
Prosocial behaviour	5.5 (2.7)	−0.07 (−1.07, 0.94)	0.03 (−1.05, 1.11)	−0.07 (−1.18, 1.03)
Total difficulties	9.3 (5.1)	2.05 (−0.07, 4.17)	1.93 (−0.25, 4.12)	1.69 (−0.48, 3.86)

^a^Bold face indicates *P < *0.05. ^b^Adjusted for child age, sex, birthweight, monthly household income, housing type, maternal education level. ^c^Model 1 additionally adjusted for maternal depressive symptoms assessed by Patient Health lgQuestionnaire-9.

Results from the complete case analyses were very similar to those of imputed data analyses (Table 3), although the associations for conduct problem were not statistically significant (*P* = 0.057 in model 3) probably due to insufficient statistical power.

For the sensitivity test, only 3.1% of mothers (8/ 253) were found to have salivary cotinine concentrations of ≥12 ng/ml. Exclusion of their children from the regression analyses (Model 3) did not change the observed associations of cotinine concentration with conduct problems (*b* = 1.05, 95% CI 0.09 to 2.02) and hyperactivity/ inattention (*b* = 1.17, 95% CI 0.12 to 2.22).

## Discussion

To our knowledge, this is the first study prospectively examining the association of SHS exposure with child behavioural problems using an objective biomarker of SHS exposure. We found that exposure to SHS during early childhood was associated with conduct problems and hyperactivity/ inattention after about a 3-year follow-up. The associations remained significant after controlling for several key confounders including birthweight, maternal education and depression^23,24^.

Comparison with earlier studies is hampered by their vastly diverse research methodologies including different tools used to assess child behavioural health. The well-established SDQ was among the most commonly used instruments in recent years, although results were inconsistent. A survey found parent-reported child SHS exposure at home was associated with SDQ-measured hyperactivity/ inattention in children^26^. Another study further showed an association with conduct problems using salivary cotinine^31^. However, neither of these studies accounted for MSP. Two cross-sectional studies found parental report of household smoking associated with child hyperactivity/ inattention, conduct and emotional problems after statistical control for MSP^32,33^, but the results were not replicated in a prospective cohort using similar measures^34^. Other prospective studies using maternal-reported measures of postnatal SHS exposure did not find significant associations between exposure and behavioural problems in children of non-smoking mothers^9,10^. Our study addressed these limitations and found cotinine-measured SHS exposure in children ≤18 months was associated with conduct problems and hyperactivity/inattention. The results were consistent with other cross-sectional studies which, after adjusting for MSP, found cotinine-measured SHS to be linked with child behavioural problems^8,22,25,35^.

The validity of our findings was further strengthened by excluding mothers who smoked to preclude the potential effect of MSP^2,5^. Self-reported smoking status by Chinese mothers with young children in Hong Kong has been validated previously^36^. Our findings did not change after restricting the analyses to children whose mothers were non-smoking with biochemically validation. Although we were unable to separate mothers who were former smokers from never-smoking mothers in our data, MSP is rare in Hong Kong (3.4%)^13^. It is also very unlikely that mothers who formerly smoked during pregnancy subsequently participated in our study, since few mothers who continued smoking during pregnancy quit after delivery of the newborn^2^.

Our study accounted for maternal depressive symptoms, which contribute to both child SHS exposure and behavioural outcomes. We observed a strong bivariable associations between maternal depressive symptoms and child behavioural problems. However, it is also possible that child behavioural problems may contribute to or aggravate maternal mental health problems. Further research using a longitudinal design and repeated measurements of child and maternal mental health is warranted.

The biological mechanisms by which postnatal SHS may contribute to child behavioural problems has remained an area of active research. Gospe and colleagues first showed the independent biological effect of postnatal SHS on neurodevelopment in rodent models: postnatal SHS exposure reduced cellular density and increased cell size in the brains of rats unexposed to tobacco smoke prenatally^37^. More recent studies have suggested exposure to nicotine in SHS leads to disruption of the cholinergic system, which may underlie the development of child behavioural problems^38^. Epigenetic processes have also been suggested to mediate the relationship between SHS and hyperactivity/inattention, although further research is needed to test the hypothesis^39^.

This study has some limitations. First, due to incomplete data and loss to follow-up, numerous cases were excluded from the study. However, our attrition analyses using both significance tests and effect size calculations showed that all measured baseline characteristics were comparable between subjects with and without complete data on salivary cotinine and SDQ, suggesting that the risk of selection bias was likely minimal. Missing data on exposure were also imputed with multiple imputation diagnostics to increase the precision of the estimates. Complete case analyses also produced similar results. Notably, the analytic sample tended to have better, though insignificant, socioeconomic profile than the excluded sample. This might skew our observed associations toward the null since children with lower socioeconomic positions are more vulnerable to SHS exposure. Second, data on child behavioural health was maternal-reported. However, the parental version of SDQ has been validated to screen for conduct disorder and ADHD in preschoolers and predicts clinical endpoints 2 years later^20^. The parental-report version of SDQ in Chinese has also been validated locally^19^. Third, although we adjusted for several key confounders, unmeasured confounding by inherited factors and residual confounding by parental and family environmental factors remain a possibility. Nevertheless, known genetic variants that predict child behavioural problems are relatively rare with weak effect sizes^40^. Finally, while our study accounted for MSP, we were not able to rule out the potential effect of maternal exposure to SHS during pregnancy on child behavioural problems^41^.

In conclusion, SHS exposure during early childhood was associated with conduct problems and hyperactivity/inattention at preschool age after precluding MSP. Young children typically reside at home most of the time throughout early childhood and are unable to protect themselves from passive smoking. Our findings, if replicated by further studies, can be a new warning for parents to quit smoking or to establish smoke-free home rules, and back implementation of smoke-free policy to safeguard the health of children.

## Data Availability

The dataset generated during and/or analysed during the current study are available from the corresponding author on reasonable request.
